# In Vivo Monitoring of Corneal Dendritic Cells in the Subbasal Nerve Plexus during Trastuzumab and Paclitaxel Breast Cancer Therapy—A One-Year Follow-Up

**DOI:** 10.3390/diagnostics12051180

**Published:** 2022-05-09

**Authors:** Sebastian Bohn, Nadine Stache, Karsten Sperlich, Stephan Allgeier, Bernd Köhler, Andreas Bartschat, Ha-Vy Do, Christian George, Rudolf F. Guthoff, Angrit Stachs, Oliver Stachs, Katharina Anna Sterenczak

**Affiliations:** 1 Department of Ophthalmology, Rostock University Medical Center, 18057 Rostock, Germany; sebastian.bohn@uni-rostock.de (S.B.); nadine.stache@uni-rostock.de (N.S.); karsten.sperlich@uni-rostock.de (K.S.); ha-vy.do@med.uni-rostock.de (H.-V.D.); rudolf.guthoff@med.uni-rostock.de (R.F.G.); oliver.stachs@uni-rostock.de (O.S.); 2 Department Life, Light & Matter, University of Rostock, 18059 Rostock, Germany; 3 Department of Obstetrics and Gynecology, University of Rostock, 18059 Rostock, Germany; christian.george@kliniksued-rostock.de (C.G.); angrit.stachs@uni-rostock.de (A.S.); 4 Institute for Automation and Applied Informatics, Karlsruhe Institute of Technology, 76021 Karlsruhe, Germany; stephan.allgeier@kit.edu (S.A.); bernd.koehler@kit.edu (B.K.); andreas.bartschat@partner.kit.edu (A.B.)

**Keywords:** paclitaxel and trastuzumab, chemotherapy-induced peripheral neuropathy (CIPN), in vivo large-area confocal laser scanning microscopy (CLSM) of the cornea, breast cancer, one-year follow-up, corneal subbasal nerve plexus (SNP), identical areas, corneal nerves, dendritic cells

## Abstract

Paclitaxel and trastuzumab have been associated with adverse effects including chemotherapy-induced peripheral neuropathy (CIPN) or ocular complications. In vivo confocal laser scanning microscopy (CLSM) of the cornea could be suitable for assessing side effects since the cornea is susceptible to, i.e., neurotoxic stimuli. The study represents a one-year follow-up of a breast cancer patient including large-area in vivo CLSM of the subbasal nerve plexus (SNP), nerve function testing, and questionnaires during paclitaxel and trastuzumab therapy. Six monitoring sessions (one baseline, four during, and one after therapy) over 58 weeks were carried out. Large-area mosaics of the SNP were generated, and identical regions within all sessions were assigned. While corneal nerve morphology did not cause alterations, the number of dendritic cells (DCs) showed dynamic changes with a local burst at 11 weeks after baseline. Simultaneously, paclitaxel treatment was terminated due to side effects, which, together with DCs, returned to normal levels as the therapy progressed. Longitudinal in vivo CLSM of the SNP could complement routine examinations and be helpful to generate a comprehensive clinical picture. The applied techniques, with corneal structures acting as biomarkers could represent a diagnostic tool for the objective assessment of the severity of adverse events and the outcome.

The incidence of cancer along with long-term survival is increasing, and future cancer therapies will reflect individually tailored therapy regimens, adjusted to the combination of the characteristics of the tumor and the patient’s response to therapy and quality of life (QOL) [1,2]. Currently, one major drawback of higher patient survival is the increase in therapy-induced adverse events such as long-lasting painful neuropathies or ocular complications.

Chemotherapy-induced peripheral neuropathy (CIPN) is one of the most frequent side effects with a prevalence of 19% to 85% [3]. There is hardly a cytostatic agent that does not exercise a side effect on the peripheral nervous system, and the longest nerves show the greatest vulnerability [4]. Further, the underlying pathological mechanisms are manifold, as cancer therapy frequently involves drug combinations, and the neurotoxic mechanisms also differ between the anti-cancer drugs [4].

Regarding breast cancer, its diagnosis and treatment are drastic events for affected women and their social lives. According to the World Health Organization (WHO) in 2020, there were 2.3 million women diagnosed with breast cancer and 685,000 deaths globally [5]. Further, the WHO states that as of the end of 2020, there were 7.8 million women alive who were diagnosed with breast cancer in the past 5 years, making it the world’s most prevalent cancer [5]. During breast cancer therapy, 75% of the patients are affected by severe side effects, particularly by CIPN, often requiring the adaption or even termination of the therapy regimen and significantly limiting the patient’s QOL. Currently, there is no approved treatment for CIPN, and early and objective detection methods are needed to implement treatment changes [6].

Taxanes, which have been approved for the treatment of various cancer types including breast cancer, show a high incidence of CIPN, ranging from 11% to 87%, with the highest rate reported for paclitaxel [3]. Taxane-induced peripheral neuropathy causes an acute, length-dependent distal sensory neuropathy, accompanied by neuropathic pain in a glove-and-stocking distribution [7]. The underlying main mechanisms of taxane-caused nerve injury include the alteration of microtubule dynamics, mitochondrial dysfunction, and oxidative stress in peripheral nerves [8]. The induced nerve injury is then followed by peripheral and central inflammation and changes in the activity of ion channels, leading to peripheral neuropathy [8]. Although the mechanisms of the damage are not completely understood, the role of neuroinflammation is becoming increasingly evident [9,10]. The accumulation of chemotherapeutical agents in dorsal root ganglia may cause neuroinflammation through activation of immune-like cells followed by secretion of mediators enhancing neuronal excitability and generating pain and hypersensitivity [9].

The cornea is considered to be one of the most densely innervated tissues in the human body, and corneal nerves, which arise from the ophthalmic branch of the trigeminal nerve, are considered part of the peripheral nervous system [11]. The cornea represents a unique model for confocal laser scanning microscopy (CLSM) based on studying neural and immune changes because of its clarity and easy accessibility for non-invasive in vivo imaging. Thus, a new approach to assess neuropathies and neuroinflammatory processes could be the analysis of the nerves and immune cells such as dendritic cells (DCs) of the eye. However, in the past, there has been limited research on corneal changes associated with neurotoxicity and even less on the potential neuropathic effects of neurotoxic chemotherapy on the ocular surface [6]. Consequently, structured and longitudinal CLSM analysis of the patient’s cornea before, during, and after cancer therapy may be a key in aiding to fill the diagnostic gap of early-stage CIPN and neurotoxicity detection. Moreover, individual follow-up monitoring of corneal nerves and DCs before, during, and after treatment could have a supportive character for the attending clinicians as cancer and therapy differ from patient to patient. In this report, we were able to identify the same corneal regions over one year using in vivo large-area CLSM and represent the first longitudinal analysis of the subbasal nerve plexus (SNP) of a human epidermal growth factor receptor (HER)-2-positive breast cancer patient before, during, and after paclitaxel and trastuzumab therapy.

In a former report, we analyzed the SNP of this patient at baseline and during the first 11 weeks after baseline monitoring [12]. While the subbasal nerve morphology was not altered significantly, a change in the number of DCs and a local burst at 11 weeks after baseline examination were detected, indicating treatment-mediated corneal inflammatory processes but a lack of neurotoxic effects [12]. Within the present report, the monitoring protocol was continued and extended beyond the therapy regimen until 58 weeks after baseline examination. To our knowledge, this is the first longitudinal study covering the monitoring of a patient’s SNP before, during, and after breast cancer chemotherapy. The monitoring sessions included in vivo CLSM of the corneal subbasal nerve plexus (SNP), general ophthalmological examinations, nerve function testings, and questionnaires to assess the patient’s symptoms and functional limitations related to CIPN. The large-area in vivo mosaic CLSM imaging of the SNP was performed according to [12]. Ophthalmological examinations included, i.e., measurement of the intraocular pressure, visual acuity, slit lamp examination of the cornea, corneal esthesiometry (Cochet–Bonnet), and retinal optical coherence tomography. Nerve function sensitivity was evaluated using the neuropathy symptom score (NSS) and neuropathy deficit score (NDS) analogous to [12,13]. CIPN symptoms and QOL from the patient’s perspective were assessed by completing the European Organisation for Research and Treatment of Cancer Quality of Life Questionnaire (EORTC QLQ)-CIPN20 and Functional Assessment of Cancer Therapy (FACT) —Taxane questionnaires [14,15].

Analogous to [12], the applied in vivo large area CLSM allowed longitudinal monitoring of the patient’s SNP over a time period of 58 weeks and allowed for the identification of a common repeatedly imaged area throughout all monitoring sessions by identification of recurrent k-structures (Figure 1). The developed and applied technique allowed large-area SNP imaging when compared to conventional CLSM methods (2.5–4.2 mm^2^ versus 0.16 mm^2^).

In line with our first results [12], SNP morphology remained stable throughout the whole monitoring period, reflecting no neurotoxic effects on corneal nerves (Figure 1). Further, DCs could be monitored and quantified over time within identical SNP areas (Figure 1 and Figure 2). At 6 and 11 weeks after baseline examination, a local burst of increasing DC numbers was detected, which decreased to normal DC levels during the downstream follow-up sessions (Figure 1 and Figure 2). In our former report, we hypothesized that this regional burst within the first 11 weeks of treatment indicated ongoing inflammation or immune-mediated reactions that might have been caused by paclitaxel or trastuzumab alone or the combination of both [12]. At 11 weeks after baseline, the patient developed severe side effects and the treatment with paclitaxel was terminated, while trastuzumab was maintained. Remarkably, after cessation of paclitaxel, the number of DCs returned to the baseline level (Figure 1 and Figure 2), and the side effects decreased, which was also reflected by the NDS and NSS (Figure 2). The EORTC QLQ-CIPN20 and FACT-Taxane questionnaires showed fluctuations that seem to be independent of the discontinuation of paclitaxel after 11 weeks of therapy and might be affected by social/emotional effects during and after breast cancer therapy (Figure 2). The performed ophthalmological examinations (Table 1) did not indicate any abnormalities.

**Figure 1:**
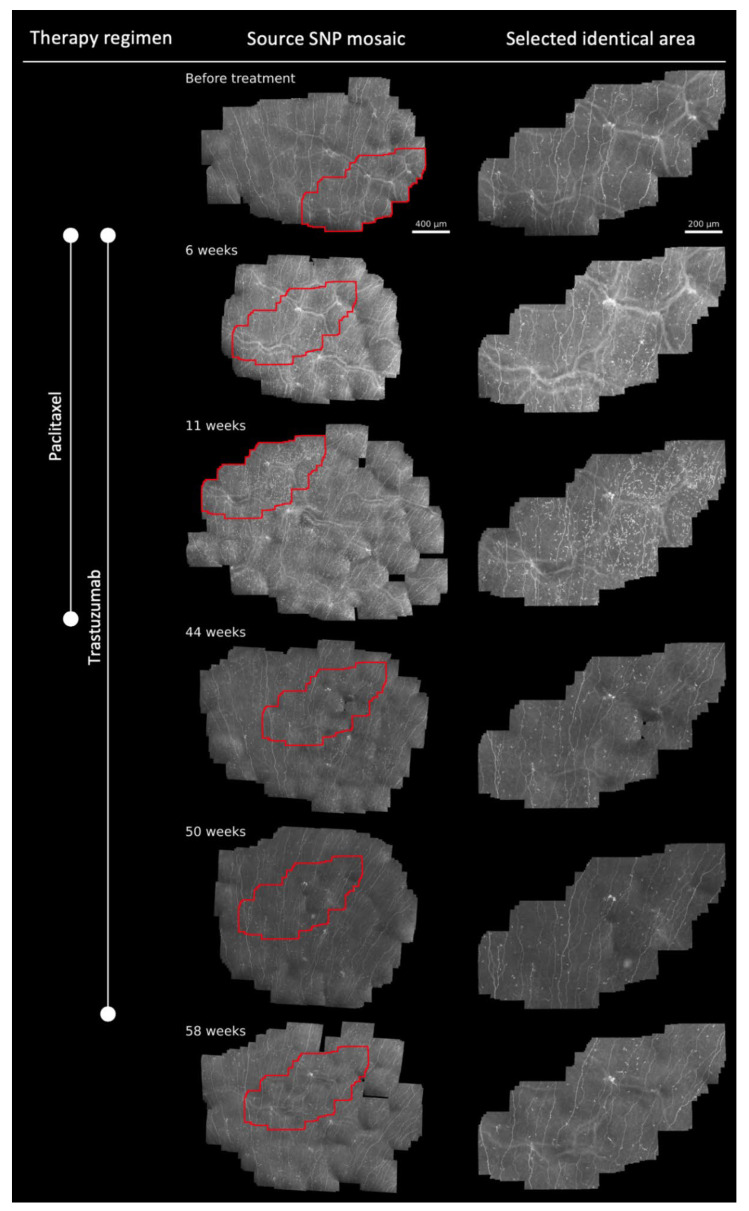
In vivo large-area confocal laser scanning microscopy (CLSM) of the corneal subbasal nerve plexus (SNP) of a breast cancer patient (human epidermal growth factor receptor (HER)-2-positive) at baseline, during, and after paclitaxel and trastuzumab therapy regimen. The detailed patient history and therapy regimen were previously described in [12]. The patient underwent in vivo large-area CLSM at 6 sessions, with a subsequent morphometric assessment of the SNP according to [12]. **First column from top to bottom**: Timeline of the cancer therapy regimen. The examinations were performed at baseline before treatment, during treatment (at 6 and 11 weeks after baseline examination with combined paclitaxel and trastuzumab therapy; at 44 and 50 weeks after baseline examination with trastuzumab-only therapy), and after treatment (58 weeks after baseline session, 4 weeks after termination of therapy). Paclitaxel therapy was terminated 11 weeks after baseline monitoring due to side effects. **Second column from top to bottom**: The patient’s SNP before treatment (at baseline), at 6 weeks, at 11 weeks, at 44 weeks, and at 50 weeks (after baseline) during treatment, and at 58 weeks after baseline monitoring (4 weeks after termination of the therapy). The recorded image data were used to generate mosaic SNP images with sizes ranging from 2.5 to 4.2 mm^2^, analogous to [16]. At baseline and at 6 and 11 weeks after baseline, the same source SNP mosaics were used as in [12]. The assignment of identical areas within all generated mosaics at different sessions was performed in all SNP source mosaics by utilizing so-called k-structures as previously applied in [12], enabling the comparison of the same areas with each other over time (red marked zones). **Third column from top to bottom**: Enlarged view of identical red marked areas. The size of the identical areas within all mosaics was 0.72 mm^2^. Nerve fiber morphology remained stable except for minor fluctuations, while dendritic cell numbers showed regional increases at 6 and 11 weeks after the baseline monitoring within selected identical areas.

**Figure 2:**
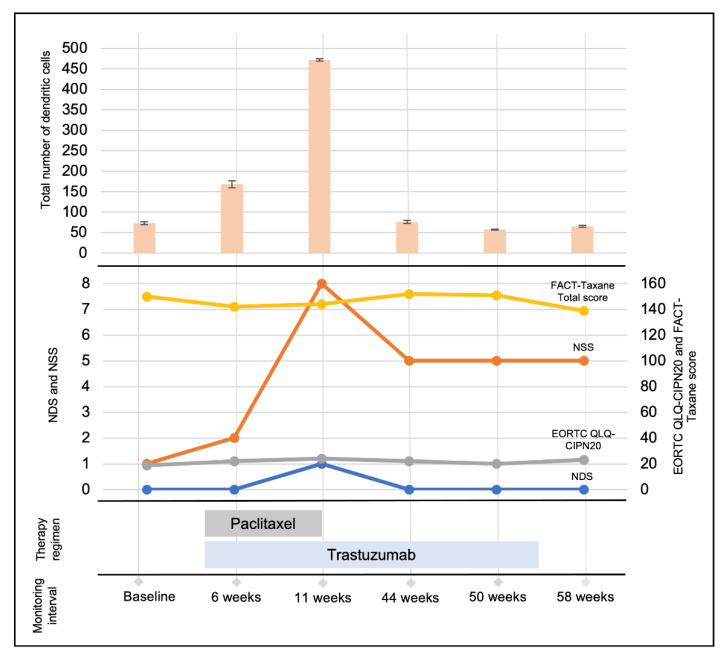
Total dendritic cell (DC) count within identical corneal areas, nerve function testing score, and questionnaires outcome of a breast cancer patient (human epidermal growth factor receptor (HER)-2-positive) in the course of paclitaxel and trastuzumab breast cancer therapy regimen and monitoring intervals. **Total number of dendritic cells:** Total DC count within identical areas of the corneal subbasal nerve plexus before therapy at baseline, during therapy (6, 11, 44, and 50 weeks after baseline monitoring), and after therapy at 58 weeks after baseline monitoring. The identical areas throughout all analyzed time points were determined analogous to [12] and accounted for 0.72 mm^2^. The number of DCs was analyzed manually in triplicate using the ImageJ Cell Counter plugin [17]. At baseline, the DC number was 73 (±3.56), at 6 weeks 167.67 (±8.34), at 11 weeks 472 (±2.94), at 44 weeks 76.33 (±4.1), at 50 weeks 57.33 (±1.25), and at 58 weeks after baseline monitoring number was 65.33 (±2.62). At 6 and 11 weeks, increasing DC numbers were detected, which subsequently decreased to normal levels during the downstream follow-up sessions after cessation of paclitaxel therapy. **Neuropathy deficit score (NDS), neuropathy symptom score (NSS), European Organisation for research and Treatment of Cancer Quality of Life Questionnaire-Chemotherapy-Induced Peripheral Neuropathy (EORTC QLQ-CIPN)20, and Functional Assessment of Cancer Therapy (FACT)-Taxane questionnaires:** Outcome of nerve function testing and questionnaires during a period of 58 weeks. Nerve function sensitivity was evaluated using NDS and NSS analogous to [13]. While the NSS testing included specific questions regarding neurological symptoms (sensory, motor) with attention to neuropathic pain occurrence and thermoregulatory disturbance, the NDS assessment included measurement of vibration and pain sensation in the feet and legs, monofilament, temperature, and the Achilles tendon reflex [13]. The assessment of the patient’s CIPN symptoms and quality of life (QOL) was analyzed by EORTC QLQ-CIPN20 and FACT-Taxane questionnaires. The EORTC QLQ-CIPN20 is a 20-item questionnaire providing information on CIPN-related symptoms and functional limitations of patients who were exposed to potentially neurotoxic agents with scores ranging from 19 for females and 20 for males (no CIPN-related symptoms) to a maximum of 80 (CIPN related-symptoms) [14]. The FACT-Taxane questionnaire was developed to measure the health-related QOL of patients receiving taxane-containing chemotherapy and is composed of a FACT-general plus a taxane-specific subscale [15]. The FACT-Taxane yields a total score that ranges between 0 and 172, with higher scores reflecting better QOL as well as individual subscale scores such as physical, social, emotional, or functional well-being. [15]. The taxane-specific subscale assesses symptoms related to neurotoxicity, arthralgia, myalgia, and skin discoloration [15]. The NDS was 0 for all monitoring sessions except at 11 weeks after baseline, where it was 1 (no peripheral neuropathy (PNP)). The NSS ranged from 1 to 8, with the highest score at week 11 (baseline: 1—no PNP; 6 weeks: 2—no PNP; 11 weeks: 8—severe PNP; 44 weeks: 5—moderate PNP; 50 weeks: 5—moderate PNP; 58 weeks: 5—moderate PNP). The EORTC QLQ-CIPN20 score ranged between 19 and 24 throughout the whole study period, with the highest level at 11 weeks after baseline monitoring (baseline: 19; 6 weeks: 22; 11 weeks: 24; 44 weeks: 22; 50 weeks: 20; 58 weeks: 23). The FACT-Taxane Total score ranged between 139 and 152, with the highest levels at week 44 (baseline: 150; 6 weeks: 142; 11 weeks: 144; 44 weeks: 152; 50 weeks: 151; 58 weeks: 139). **Therapy regimen:** Combined paclitaxel and trastuzumab therapy started 4 weeks after baseline monitoring. Paclitaxel treatment was discontinued at 11 weeks after baseline monitoring due to side effects, while trastuzumab was maintained until 54 weeks after baseline. **Monitoring interval:** A total of six monitoring sessions (one at baseline, four during, and one after therapy) over 58 weeks were carried out. Please note the non-equidistant horizontal time axis.

In a recent study [18], corneal nerve morphology was investigated in patients who have completed neurotoxic chemotherapy with either paclitaxel or oxaliplatin well after treatment cessation. In a sub-analysis, the differences in corneal nerve parameters of paclitaxel-treated patients with neuropathy and without neuropathy in comparison to healthy controls were explored [18]. Corneal nerve parameters, i.e., corneal nerve fiber length and nerve fiber density, were significantly reduced in paclitaxel-treated patients with neuropathy compared with healthy controls and paclitaxel-treated patients without neuropathy, whereas the latter had similar levels to healthy controls [18]. According to the authors, the findings implied that corneal nerve dysfunction is more evident in patients with CIPN, supporting the utility of CLSM techniques for monitoring nerve function in patients receiving paclitaxel [18]. These results are also reflected by the present study as the morphology of the corneal nerves did not change over time and the patient did not develop CIPN.

In another study, the SNP and corneal DCs were analyzed 3 to 24 months following neurotoxic treatment with oxaliplatin and paclitaxel [19]. While corneal nerve parameters were reduced, there were no significant changes in DC count in paclitaxel-treated participants [19]. The DC count in the present study showed comparable results at baseline and at the end of therapy. Despite the burst of DCs between 6 and 11 weeks after baseline monitoring, the DC numbers returned to normal levels until therapy cessation and beyond (Figure 1 and Figure 2). Moreover, within the present study, identical SNP regions were examined over time. Consequently, therapy-induced effects could be directly compared with each other during the course of therapy, representing the first longitudinal study with a constant examination of identical SNP areas over time. Nevertheless, the underlying mechanisms leading to the regional DC burst are still unclear and remain open for discussion within the community, and possible reasons have already been discussed in [12]. One further reason could be an interaction between paclitaxel and trastuzumab during the combined administration as the effects diminished after the termination of paclitaxel. However, within the present study, the analysis of DCs is limited to total DC counts without distinction between mature and immature types. Currently, we do not have a tool for the differentiation between both types, and there is a strong need to develop clear, quantitative criteria including cell size and shape for future studies. Furthermore, the herein reported results demonstrate that corneal parameters such as DC number show a dynamic character, and it will be of clinical value to analyze such dynamic parameters within a defined corneal region by using the presented technique in a larger cohort of patients undergoing paclitaxel and trastuzumab and other chemotherapy regimens over time. Further, early changes within the SNP could be monitored even more closely in order to adapt therapy regimens before the occurrence of quality of life-limiting side effects. Nevertheless, we have to conclude that the same areas analyzed were small compared to the size of the source SNP mosaics. In future studies, new technologies for real-time area location control during ongoing CLSM sessions will be needed. At the present time, the manual registration of longitudinal large-area images and analysis of the same areas is ambitious, time-consuming, and needs advanced expertise.

In conclusion, the key breakthrough of the present study is that the applied technology opens the window for monitoring of identical corneal SNP areas, which is of great clinical benefit for long-term studies in the setting of neuropathy. In vivo CLSM monitoring of the patient’s cornea could complement routine examinations and be helpful in the generation of a more comprehensive clinical picture. Regarding the future trend with personalized medicine and individually tailored cancer therapy, large-area CLSM imaging could represent a diagnostic tool for the objective assessment of the severity of adverse events and the outcome for the patient with corneal nerves and DCs acting as potential biomarkers.

## Figures and Tables

**Table 1:** Summary of ophthalmological examinations at baseline, and at 6, 11, 44, 50, and 58 weeks after baseline monitoring. The conducted examinations included measurement of the intraocular pressure (IOP), visual acuity, slit lamp examination of the cornea, corneal esthesiometry (Cochet–Bonnet), and retinal optical coherence tomography.

	Baseline	6 Weeks	11 Weeks	44 Weeks	50 Weeks	58 Weeks
**IOP (mmHg)**	18	17	17	18	21	17
**UCVA (logMAR)**	0.4	0.4	0.4	0.4	0.4	0.4
**Slit lamp**	regular	regular	swelling of conjunctiva	regular	regular	regular
**Corneal esthesiometry**	regular (60 mm)	regular (60 mm)	regular (60 mm)	regular (60 mm)	regular (60 mm)	regular (60 mm)
**Retina OCT**	regular	regular	regular	regular	regular	regular

IOP = intraocular pressure; UCVA = uncorrected visual acuity; logMAR = logarithm of the minimum angle of resolution; OCT = optical coherence tomography.

## Data Availability

The data presented in this study are available on request from the corresponding author.
